# Mortality Risk and Antibiotic Therapy for Patients with Infections Caused by *Elizabethkingia* Species—A Meta-Analysis

**DOI:** 10.3390/medicina60091529

**Published:** 2024-09-19

**Authors:** Chienhsiu Huang, Sufang Kuo, Lichen Lin

**Affiliations:** 1 Department of Internal Medicine, Dalin Tzu Chi Hospital, Buddhist Tzu Chi Medical Foundation, No. 2, Min-Sheng Road, Dalin Town, Chiayi 62247, Taiwan; 2 Department of Nursing, Dalin Tzu Chi Hospital, Buddhist Tzu Chi Medical Foundation, No. 2, Min-Sheng Road, Dalin Town, Chiayi 62247, Taiwan; dl08596@tzuchi.com.tw (S.K.); df462594@tzuchi.com.tw (L.L.)

**Keywords:** *Elizabethkingia* species infection, mortality risk, comorbidity, antibiotic treatment, inappropriate antimicrobial therapy

## Abstract

*Background and Objectives*: Patients with infections caused by *Elizabethkingia* species require prompt identification and effective antibiotic treatment since these spp. are typically resistant to multiple antibiotics and variable susceptibility patterns. Understanding the mortality risk of this disease is difficult because of the relatively low incidence of infections caused by *Elizabethkingia* spp. and the lack of published systematic evaluations of the risk factors for mortality. The aim of the present study was to investigate risk factors for mortality in patients with infections caused by *Elizabethkingia* spp. by conducting a meta-analysis of existing studies on these infections. *Materials and Methods*: Studies comparing patients who died from infections caused by *Elizabethkingia* spp. with patients who survived were considered for inclusion. Studies that reported one or more risk factors for mortality were considered. Clinical predisposing variables, predisposing comorbidities, and clinical outcomes of antibiotic treatment were among the risk factors for mortality. *Results*: The meta-analysis included twenty studies with 990 patients, and 298 patients (30.1%) died. The following risk factors for mortality were identified: intensive care unit admission, the need for mechanical ventilation, immunosuppressive or steroid therapy use, pneumonia, comorbid liver disease, and the use of inappropriate antimicrobial therapy. *Conclusions*: The use of appropriate antimicrobial therapy is critical for the effective management of infections caused by *Elizabethkingia* spp. Antimicrobial susceptibility testing would be a more reliable means of guiding treatment. The identification of the best antimicrobial drugs is needed to ensure optimal treatment recommendations for treating *Elizabethkingia*-related infections.

## 1. Introduction

### 1.1. Taxonomy and Nomenclature

*Elizabethkingia* is a genus of Gram-negative bacteria. They are frequently found in natural settings and locations, including wetlands, inland waters, hospitals, food products, plants, soil, fish, frogs, and insects [1,2]. The bacterium was first named *Flavobacterium meningosepticum* when it was identified in 1959 by Elizabeth King [3]; however, in 1994, it was suggested that the name be changed *to Chryseobacterium meningosepticum* [4]. Li Y et al. discovered a new spp. in 2003 named *Chryseobacterium miricola* [5]. On the basis of 16S rRNA phylogenetic investigations, in 2005, Kim KK et al. classified *Elizabethkingia* within the *Flavobacteriaceae* family [6]. Kämpfer et al. (2011) discovered the third spp., *Elizabethkingia anophelis*, in the midgut of Anopheles gambiae mosquitoes in Gambia, Africa [7]. *Elizabethkingia bruuniana*, *Elizabethkingia ursingii*, and *Elizabethkingia occulta* were proposed as novel members of the genus *Elizabethkingia* after Nicholson et al. (2017) investigated bacteria of the unknown Centers for Disease Control and Prevention genome spp. [8]. A novel spp. in the genus *Elizabethkingia* was discovered, for which the name *Elizabethkingia argenteiflava* spp. novel was proposed by Hwang JH et al. in 2021 [9]. Another new spp., *E. umeracha* spp. novel, was discovered by Hem S et al. in 2022 [10]. Eight spp.—*E. meningoseptica*, *E. miricola*, *E. anophelis*, *E. bruuniana*, *E. ursingii*, *E. occulta*, *E. argenteiflava*, and *E. umeracha*—now make up the genus *Elizabethkingia*.

### 1.2. Identification of Species

The treatment of human infections is impeded by challenges associated with accurately identifying *Elizabethkingia* at the spp. level via conventional techniques [11,12]. Accurate microbial identification is necessary for differentiating between *E. anophelis* and *E. meningoseptica*; however, the phenotypic similarity between the two spp. makes identification difficult. A 16S rRNA gene analysis revealed that *E. meningoseptica* and *E. anophelis* presented 98.6% similarity. This closeness has led to many misidentifications of both bacteria [13]. Systems that use a matrix-assisted laser desorption/ionization time-of-flight mass spectrometry system (MALDI-TOF MS) and have an expanded spectrum database are capable of accurately identifying *E. anophelis* and *E. meningoseptica*. A study by Sarathi S. showed that all 80 *Elizabethkingia* isolates were identified as *E. anophelis* via MALDI-TOF MS, although all of the isolates were identified as *E. meningoseptica* by the VITEK-2 compact system [14]. Seventy-nine blood isolates were investigated by Chew et al. to determine whether there were mistaken cases of *E. anophelis* among their *Elizabethkingia* spp. Each isolate was identified as either *E. meningoseptica* (96.2%) or *E. miricola* (3.8%) via the Bruker MALDI Biotyper (bioMérieux). Following nearly full-length 16S rRNA gene sequencing, 78/79 (98.7%) of the isolates were identified as *E. anophelis* [15]. Many previously recorded instances of *E. meningoseptica* may have been caused by *E. anophelis*. Thus, *E. anophelis* is considered an emerging opportunistic pathogen, and it has clinical significance. Nonetheless, *E. meningoseptica* and *E. anophelis* are the two main pathogens of the genus *Elizabethkingia* [16]. The remaining spp. of the genus cannot be successfully distinguished from *E. meningoseptica* via a MALDI-TOF MS system with modified databases. Molecular methods, such as whole-genome and housekeeping gene sequencing, are necessary for accurate spp. identification [11].

### 1.3. Epidemiology

The most common *Elizabethkingia* infection in infants is meningitis, and individuals with impaired immune systems have shown a variety of serious infections, including meningitis, bacteraemia, pneumonia, and urinary tract infections [17]. The incidence of *Elizabethkingia* spp. infection is not entirely clear. However, the prevalence of infections caused by *Elizabethkingia* spp. has increased recently in many different countries. In Wisconsin, USA, community-acquired *Elizabethkingia anophelis* caused an outbreak from 2015 to 2016. The pandemic extended to the nearby states of Illinois and Michigan, affecting a total of 66 people [18]. The acquisition of infections caused by *Elizabethkingia* spp. increased in Korea, from 0.02 cases per 1000 admissions in 2009 to 0.88 cases per 1000 admissions in 2017 [19]. The incidence of *E. meningoseptica* bacteraemia cases per 1000 admissions increased in Taiwan from 0.075 cases in 1996 to 0.356 cases in 2006 [20]. A study by Ma S et al. revealed that the incidence of infection with *E. meningoseptica* increased from 0 cases per 1000 inpatients in 2011 to 0.19 cases in 2019 [21]. Infection caused by *Elizabethkingia* spp. is a growing public health concern that should not be disregarded. Two common manifestations of *Elizabethkingia* infection reported in the literature are pneumonia and bacteraemia [22,23,24,25]. Despite existing reports of *E. anophelis*-induced meningitis, compared with *E. anophelis*, *E. meningoseptica* is more commonly linked to meningitis [26].

### 1.4. Antibiotic Resistance Genes

Infection caused by *Elizabethkingia* spp. requires careful selection of appropriate antimicrobial agents because of the intricate nature of the bacteria, which includes the development of biofilms, penetration of host cells, and resistance to multiple antibiotics. *Elizabethkingia* bacteria have inherent resistance to a wide range of β-lactam antibiotics, β-lactam/lactamase inhibitors, and carbapenems. Two different class B metallo-β-lactamases (MBLs), termed *bla*BlaB and *bla*GOB, together with a class A extended-spectrum β-lactamase (ESBL) called *bla*CME, are responsible for this resistance [27,28,29]. Furthermore, mutations in the topoisomerase IV and/or DNA gyrase genes confer resistance to fluroquinolones [30].

### 1.5. Aim of the Meta-Analysis

Fully comprehending the mortality risk of infections caused by *Elizabethkingia* spp. is difficult because of the relatively low incidence of these infections and the lack of published systematic evaluations of the risk factors for mortality. To bridge this knowledge gap, the present study aimed to investigate risk factors for mortality in patients with infections caused by *Elizabethkingia* spp. by conducting a meta-analysis of existing studies on these infections. The study explored clinical predisposing variables, predisposing comorbidities, and clinical outcomes of antibiotic treatment among the risk factors for mortality.

## 2. Materials and Methods

### 2.1. Data Search Strategy

The PubMed, Web of Science, and Cochrane Library databases were used in the literature search to find all relevant clinical trials, meta-analyses, and systematic reviews published on the subject since 1 January 2000. The phrases “*Elizabethkingia* OR *Elizabethkingia meningoseptica* OR *Elizabethkingia anophelis* or *Elizabethkingia* infection” in connection with “mortality”, “attributable mortality”, “risk factor”, “outcome”, or “prognostic factors” were searched. We searched the pertinent articles published from the beginning of the search period through 30 April 2024, and we looked at treatment trials that directly evaluated the mortality risk variables for *Elizabethkingia* spp.-deceased patients and -surviving patients. We also looked through the bibliographies of the proceedings from the retrieved publications. Only English articles were included.

### 2.2. Study Selection and Data Extraction

Two authors independently checked and evaluated each study for eligibility. After eliminating duplicates, two researchers analyzed the titles and abstracts of all of the retrieved papers to identify qualifying records. After eliminating irrelevant studies, the eligibility of all relevant papers was determined by reading their full texts. Information on the author, year of publication, country, study design, study years, total number of deceased *Elizabethkingia* spp. infection patients, total number of surviving *Elizabethkingia* spp. infection patients, clinical predisposing factors, predisposing comorbidities, and clinical outcomes of antibiotic therapy were extracted from the full-text articles. When disagreements arose, a third author resolved them.

### 2.3. Inclusion and Exclusion Criteria

In the current meta-analysis, we considered randomized controlled trials, prospective studies, and retrospective studies. Only studies that explicitly examined the mortality risk variables for *Elizabethkingia* spp. infection between patients who died and those who survived were deemed suitable for inclusion. The meta-analysis excluded patients aged less than 18 years. Studies including fewer than ten patients were also excluded.

All studies were included if they reported one or more of the mortality risk factors. The mortality risk factors included clinical predisposing factors, predisposing comorbidities, and clinical outcomes of antibiotic therapy. The clinical predisposing factors included sex, age ≥ 65 years, receiving immunosuppressive or steroid therapy, pneumonia, bacteremia, meningitis, intensive care unit (ICU) admission, and requiring mechanical ventilation. The predisposing comorbidities included malignancies, liver disease, chronic kidney disease (including end-stage renal disease), diabetes mellitus (DM), cerebral vascular disease, cardiovascular disease (excluding hypertension), and pulmonary disease. The clinical outcomes of antibiotic therapy included appropriate antimicrobial therapy, receiving a piperacillin/tazobactam-based regimen, receiving a fluoroquinolone-based regimen, and receiving a minocycline-based regimen.

### 2.4. Quality Assessment and Statistical Analysis

The Risk of Bias in Nonrandomized Studies of Interventions (ROBINS-I) tool was used to evaluate observational studies [31]. RevMan 5 and Cochrane Review Manager software (https://www.riskofbias.info/welcome/home) were used for statistical analyses. Fixed effects and random effects were utilized for data analysis. Statistical heterogeneity was evaluated using the *Q*-test and *I*^2^ statistical techniques. Significant heterogeneity between studies was defined as an *I*^2^ above 50% and a *p* value for the *Q*-test less than 0.10 for each study. We tabulated the study intervention features and compared them to the scheduled groups for each synthesis using forest plots. The funnel plot was examined to determine the degree of publication bias.

## 3. Results

### 3.1. Characteristics of the Included Trials

**Figure 1:**
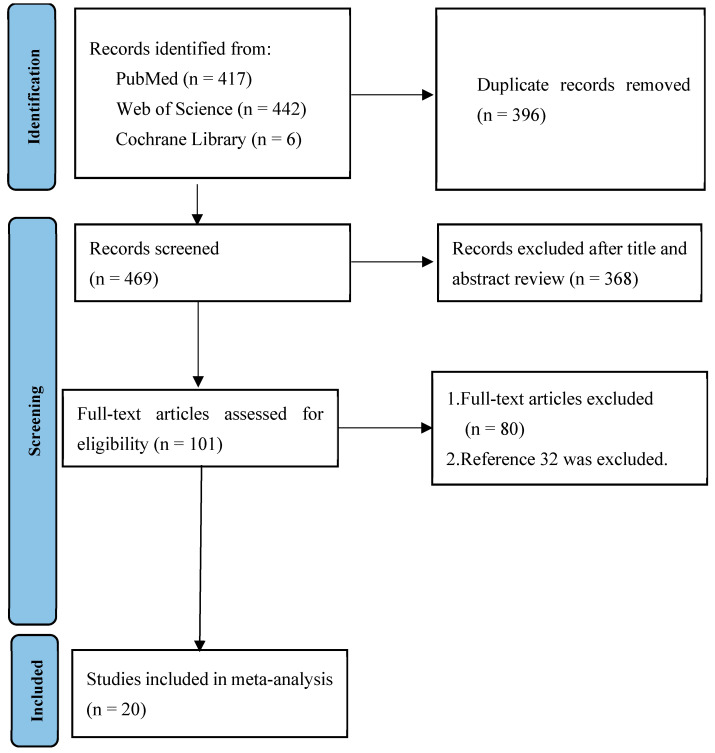
Flow diagram of the study selection process.

The details of the study selection process are shown in Figure 1. After excluding duplicates and irrelevant studies, 101 potentially relevant articles remained. After full-text article review, 80 articles were excluded because they lacked results comparing the risk factors for mortality due to infections caused by *Elizabethkingia* spp. between deceased patients and surviving patients. There are two studies by Lin JN et al. in the literature. The patients included in the two studies are identical. As a result, one study was excluded from the present meta-analysis [32]. Ultimately, 20 studies were included in the meta-analysis [14,15,19,20,21,22,24,25,33,34,35,36,37,38,39,40,41,42,43,44]. All of the included studies were retrospective studies, and their main characteristics are shown in Table 1. All of the studies had a high risk of bias (Table 2). There were 990 patients with infections caused by *Elizabethkingia* spp., including 298 (30.1%) patients who died, in the present meta-analysis.

### 3.2. Clinical Predisposing Factors

**Figure 2:**
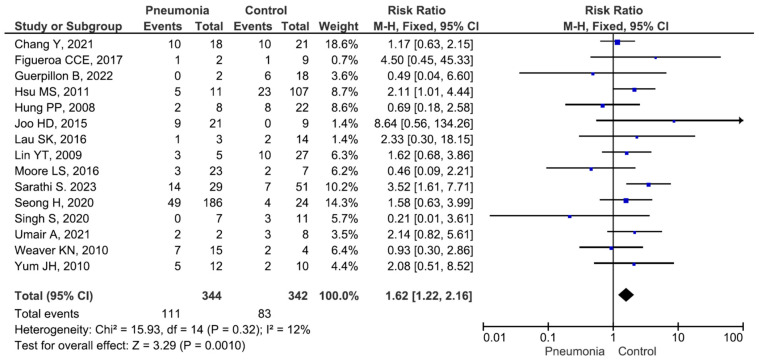
Mortality in patients with and without pneumonia [14,16,20,22,24,33,34,35,36,39,40,41,42,43,44].

**Figure 3:**
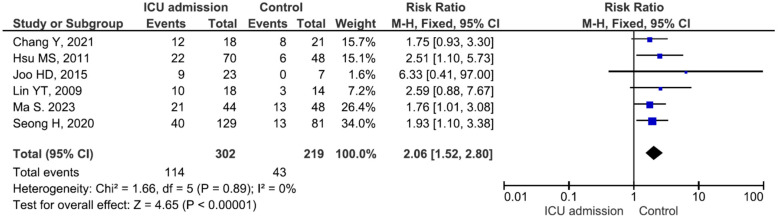
Mortality in patients with and without ICU admission [20,21,24,34,36,40].

**Figure 4:**
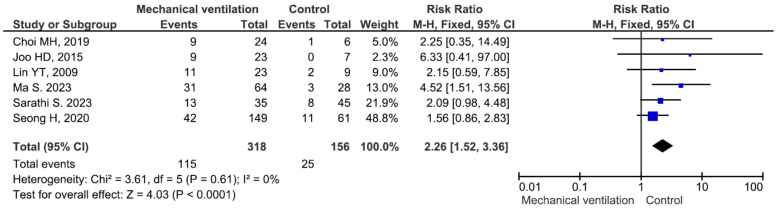
Mortality in patients with and without mechanical ventilation [14,19,21,24,36,40].

**Figure 5:**
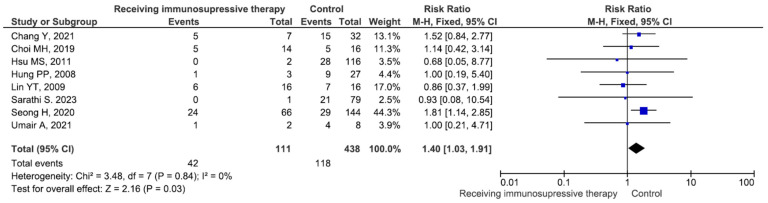
Mortality in patients receiving immunosuppressive or steroid therapy and not receiving such therapy [14,19,20,22,24,34,40,42].

There were no significant differences in the mortality rate between males and females or between patients aged ≥65 years and those aged <65 years (Figures S1 and S2). Five studies included 9 patients with meningitis (mortality rate 33.3%) and 120 patients without meningitis (mortality rate 40.0%), and the difference between the groups was not significant (RR = 0.99, *p* = 0.98) (Figure S3). Thirteen studies included 219 patients with bacteraemia (mortality rate 28.3%) and 357 patients without bacteraemia (mortality rate 28.5%), and the difference between the groups was not significant (RR = 1.05, *p* = 0.79) (Figure S4). Fifteen studies included 344 patients with pneumonia (mortality rate 32.2%) and 342 patients without pneumonia (mortality rate 24.2%). The difference was significant (RR = 1.62, *p* = 0.001) (Figure 2). Six studies included 302 patients with intensive care unit (ICU) admission (mortality rate 37.7%) and 219 patients without ICU admission (mortality rate 19.6%), and the difference between the groups was significant (RR = 2.06, *p* < 0.001) (Figure 3). Six studies included 318 patients receiving mechanical ventilation (mortality rate 36.1%) and 156 patients not receiving mechanical ventilation (mortality rate 16.0%), and the difference between the groups was significant (RR = 2.26, *p* < 0.001) (Figure 4). Eight studies included 111 patients receiving immunosuppressive or steroid therapy (mortality rate 37.8%) and 438 not receiving such therapy (mortality rate 26.9%), and the difference between the groups was significant (RR = 1.40, *p* = 0.03) (Figure 5).

### 3.3. Predisposing Comorbidities

**Figure 6:**
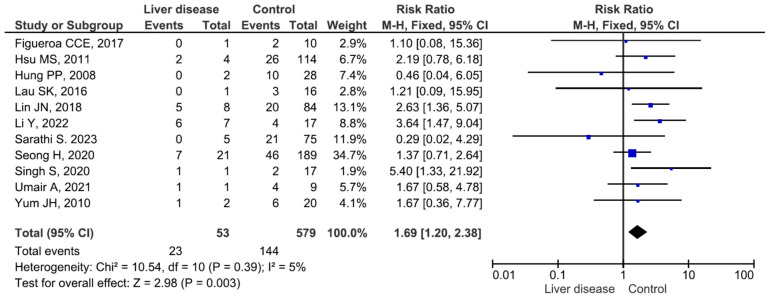
Mortality in patients with and without liver disease6 [14,16,20,22,33,37,38,40,41,42,44].

Eleven studies included 53 patients with chronic liver diseases (mainly liver cirrhosis) (mortality rate 43.3%) and 579 patients without chronic liver diseases (mortality rate 24.8%), and the difference between the groups was significant (RR = 1.69, *p* = 0.003) (Figure 6). There were no significant differences in *Elizabethkingia* spp. infection-related mortality between patients with and without cardiovascular disease, pulmonary disease, chronic kidney disease, diabetes mellitus (DM), cerebral vascular disease, or malignant disease (Figures S5–S10).

### 3.4. Antibiotic Therapy for Patients with Infections Caused by Elizabethkingia Species

**Figure 7:**
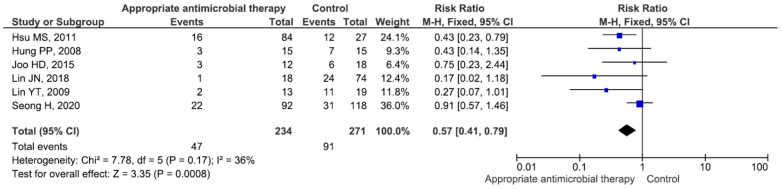
Mortality in patients receiving inappropriate and appropriate antibiotic therapy [20,22,24,36,37,40].

Six studies included 234 patients who received appropriate antibiotic therapy (mortality rate 20.0%) and 271 who received inappropriate therapy (mortality rate 33.5%), and the difference between the groups was significant (RR = 0.57, *p* < 0.001) (Figure 7). Five studies included 57 patients receiving piperacillin/tazobactam-based antibiotic therapy (mortality rate 22.8%) and 357 who did not receive such therapy (mortality rate 28.2%), and the difference between the groups was not significant (RR = 0.85, *p* = 0.50) (Figure S11). Eight studies included 164 patients who received fluoroquinolone-based antibiotic therapy (mortality rate 26.2%) and 356 patients who did not receive such therapy (mortality rate 31.1%), and the difference between the groups was not significant (RR = 0.88, *p* = 0.38) (Figure S12). Two studies included 7 patients who received minocycline-based antibiotic therapy (mortality rate 71.4%) and 42 patients who did not receive such therapy (mortality rate 47.6%), and the difference between the groups was not significant (RR = 2.97, *p* = 0.24) (Figure S13).

## 4. Discussion

The present meta-analysis of 20 studies provides evidence that the risk factors for mortality in patients with infections caused by *Elizabethkingia* spp. are ICU admission, the need for mechanical ventilation, immunosuppressive or steroid therapy use, pneumonia, comorbid liver disease (mainly liver cirrhosis), and the use of inappropriate antimicrobial therapy.

### 4.1. Clinical Factors Predisposing Patients to Mortality

Male sex (OR = 0.27, *p* = 0.031) was found to be an independent risk factor for death in patients who had *E. meningoseptica* infection in the study of Ma S et al., and this study revealed that female sex was a risk factor for death in patients with *E. meningoseptica* infection [21]. In contrast, Yum JH et al. reported on twenty-two patients with *E. meningoseptica* infection, seven of whom were male (OR = 9.0, *p* = 0.022). Male sex was a risk factor for patients with *E. meningoseptica* infection [44]. Sixteen studies in the present meta-analysis showed that sex was not a risk factor for mortality in patients with infections caused by *Elizabethkingia* spp. [14,16,19,20,21,22,33,34,35,36,37,38,39,40,41,42,43]. We concluded that sex did not affect the risk of mortality from infections caused by *Elizabethkingia* spp. In the study by Ma S et al., 34 patients died from *E. meningoseptica* infection; the 34 patients who died were much older (60.88 ± 16.27 versus 52.21 ± 17.97 years) (OR = 1.04, *p* = 0.019) than survivors were [21]. Twelve studies in the present meta-analysis demonstrated that there was no correlation between being older than 65 years and a higher risk of mortality [16,21,22,24,33,35,37,39,41,42,43,44]. We deduced that infections caused by *Elizabethkingia* spp. did not increase the risk of mortality in patients older than 65 years. The use of immunosuppressive agents or steroids in relation to the mortality risk of patients with infections caused by *Elizabethkingia* spp. has not been extensively studied. On the basis of the present meta-analysis, patients with infections caused by *Elizabethkingia* spp. infection were at increased risk of mortality if they were receiving therapy with immunosuppressive agents or steroids. The risk of mortality may be affected by the use of immunosuppressive agents or steroids, which compromise the immune status of patients.

In the present meta-analysis, six studies reported that 318 patients with infections caused by *Elizabethkingia* spp. needed mechanical ventilation: all six studies reported a trend towards a high mortality risk for patients with infections caused by *Elizabethkingia* spp. who need mechanical ventilation [14,19,21,24,36,40]. Ma S et al. reported that 34 deceased patients with *E. meningoseptica* infection had a significantly greater need for mechanical ventilation (OR = 9.51, *p* = 0.004) [21]. In a study by Sarathi S et al., 35 patients needed mechanical ventilation, and the mortality rate was 37.1% (OR = 3.84, *p* = 0.013). The need for mechanical ventilation was reported to be an independent risk factor for mortality in patients with bloodstream infection caused by *Elizabethkingia* spp. [14]. With respect to ICU admission, six studies included 302 patients with infections caused by *Elizabethkingia* spp. who were admitted to the ICU: all six studies revealed a trend towards a high risk of mortality for patients with infections caused by *Elizabethkingia* spp. who are admitted to the ICU [20,21,24,34,36,40]. Hsu MS et al. reported that 22 decreased patients with *E. meningoseptica* bacteraemia had a significantly greater need for ICU admission (OR = 2.51, *p* = 0.027) [20]. In our meta-analysis, ICU admission and the need for mechanical ventilation were associated with increased mortality risk. This emphasizes how crucial it is to account for how serious the underlying risk factors are when determining a patient’s prognosis for *Elizabethkingia* spp. infection.

Thirteen studies in the present meta-analysis demonstrated that bacteraemia was not associated with an increased risk of mortality [16,20,22,24,33,34,35,39,40,41,42,43,44]. Five studies in the present meta-analysis demonstrated that meningitis was not associated with an increased risk of mortality [22,24,34,41,42]. There is no evidence in the literature that bacteraemia and meningitis are risk factors for mortality in patients with infections caused by *Elizabethkingia* spp. We concluded that bacteraemia and meningitis were not risk factors for mortality in patients with infections caused by *Elizabethkingia* spp. With respect to pneumonia, fifteen studies reported 686 cases of pneumonia caused by *Elizabethkingia* spp. [14,16,20,22,24,33,34,35,36,39,40,41,42,43,44]. Ten studies revealed a trend towards a high risk of mortality in patients with pneumonia caused by *Elizabethkingia* spp. [14,16,20,24,33,34,36,40,42,44]. Pneumonia (OR = 8.64, *p* = 0.029) was found to be a risk factor for mortality in patients who were infected with *E. meningoseptica* by Joo HD et al. [36]. Pneumonia (OR = 8.58, *p* < 0.001) was demonstrated to be a risk factor for mortality in patients with infections caused by *Elizabethkingia* spp. by Sarathi S et al. [14]. Patients with pneumonia are more likely to develop respiratory failure than those with bacteraemia and meningitis. Pneumonia and the need for mechanical ventilation are associated with mortality risk. Four studies simultaneously revealed that pneumonia and the need for mechanical ventilation were risk factors for mortality in patients with infections caused by *Elizabethkingia* spp. [14,24,36,40]. We concluded that pneumonia was a risk factor for mortality in patients with infections caused by *Elizabethkingia* spp. in the present meta-analysis, which is consistent with the literature.

### 4.2. Comorbidities Predisposing Patients to Mortality

Few studies have explored the comorbidities that predispose patients with infections caused by *Elizabethkingia* spp. to mortality. In a univariate analysis, Lin JN et al. showed that the mortality rate was greater in individuals with liver cirrhosis (OR = 2.63, *p* = 0.032) [37]. Li Y et al. reported that seven patients with *E. meningoseptica* infection had liver disease, and the mortality rate was 85.7% (OR = 3.64, *p* = 0.009). Comorbid liver disease was an independent risk factor for mortality in patients with *E. meningoseptica* infection [38]. In the present meta-analysis, only liver disease, especially liver cirrhosis, was associated with mortality in patients with infections caused by *Elizabethkingia* spp.

With respect to other comorbidities, twelve studies reported on 157 patients with infections caused by *Elizabethkingia* spp. and DM: ten of these twelve studies reported that DM was not a risk factor for mortality in patients with infections caused by *Elizabethkingia* spp. [16,20,21,22,33,35,38,40,41,44], and two (Sarathi S and Umair A) reported that DM was a risk factor for mortality in patients with infections caused by *Elizabethkingia* spp. [14,42]. Sarathi S et al. reported that there were twenty infected patients with DM, and the mortality rate was 45% (OR = 4.09, *p* = 0.01). DM is an independent risk factor for mortality in patients with bloodstream infections caused by *Elizabethkingia* spp. [14]. Umair A et al. reported that six patients had comorbid DM, and the mortality rate was 83.3% (OR = 7.86, *p* = 0.048). Therefore, DM is also an independent risk factor for mortality in patients with infections caused by *E. meningoseptica* [42]. Joo HD et al. reported eight patients with end-stage renal disease, and the mortality rate was 62.5% (OR = 3.44, *p* = 0.032). End-stage renal disease is an independent risk factor for mortality in patients with *E. meningoseptica* infection [36]. According to a study by Chang Y et al., univariate analysis revealed that a higher mortality rate with *E. anophelis* infection was linked to both chronic obstructive pulmonary disease (OR = 10.60, *p* = 0.020) and cerebrovascular disease (OR = 3.05, *p* = 0.035) [34]. Insufficient studies have reported DM, end-stage renal disease, chronic obstructive pulmonary disease, and cerebrovascular disease related to mortality in patients with *Elizabethkingia* spp. infection. These few studies do not support that DM, end-stage renal disease, chronic obstructive pulmonary disease, and cerebrovascular disease are risk factors for mortality in patients with *Elizabethkingia* spp. infection. We concluded that no comorbidities other than liver disease were risk factors for mortality in patients with infections caused by *Elizabethkingia* spp.

### 4.3. Antibiotic Therapy for Infections Caused by Elizabethkingia Species

Ma S et al.‘s systematic evaluation revealed that the risk factor for mortality in patients with *E. meningoseptica* infection most frequently mentioned in the literature is inappropriate antibiotic treatment [21]. The only predictor linked to a 14-day survival rate in the multivariate analysis of risk factors was appropriate definite antibiotic use (OR = 0.11, *p* = 0.007), according to the study of Huang YC et al. [45]. Lin YT et al. suggested that the primary risk factor for mortality in patients with *E. meningoseptica* bacteraemia was the use of inappropriate antibiotics (OR = 3.76, *p* = 0.028) [24]. In a multivariate analysis, Lin JN et al. reported that inappropriate empirical antimicrobial therapy was a risk factor for mortality in patients with infections caused by *Elizabethkingia* spp. (OR = 12.45, *p* = 0.027) [37]. In the present meta-analysis, six studies reported on appropriate antimicrobial therapy. All of the studies revealed that appropriate antimicrobial therapy was associated with lower mortality risk [20,22,36,37,38,40]. We concluded that patients with infections caused by *Elizabethkingia* spp. who receive inappropriate antimicrobial therapy have an increased mortality risk.

Our meta-analysis evaluated piperacillin/tazobactam-based, fluoroquinolone-based, and minocycline-based therapies, and there were no significant differences in mortality among the groups. The factors affecting the outcome of antibiotic therapy are underlying medical conditions, the appropriateness of antimicrobial therapy, and the antimicrobial susceptibility patterns of *Elizabethkingia* spp. In addition, few studies have explored this issue regarding antimicrobial therapies, especially minocycline-based therapy. With respect to minocycline, there were four studies reported in the literature in which the antimicrobial susceptibility to minocycline was greater than 90%. Minocycline was effective against *Elizabethkingia* spp. isolates [37,46,47,48]. We could not further analyze the impact of these factors on the risk of mortality. The present meta-analysis could not draw conclusions related to the mortality risk of antibiotic therapy regimens in patients with infections caused by *Elizabethkingia* spp.

### 4.4. Combination Antibiotic Therapy for Infections Caused by Elizabethkingia Species

Seong et al.‘s study of patients with infections caused by *Elizabethkingia* spp. included 187 patients receiving combination treatment (mortality rate: 25.6%) and 23 patients receiving monotherapy (mortality rate: 21.7%). The difference between the groups was not significant (OR = 0.84, *p* = 0.8) [40]. Chan JC et al. reported that in 13 pediatric patients with *E. meningoseptica* infections, treatment with combination therapy consisting of trimethoprim-sulfamethoxazole and piperacillin/tazobactam or fluoroquinolone was successful (cure rate of 81.8%) [49]. Tang HJ et al. investigated the in vitro efficacy of antibiotics against clinical isolates of *Elizabethkingia anophelis*. The study revealed that the combination of minocycline and rifampicin had the greatest synergistic effect on 93.3% of the isolates; no antagonistic effects were observed. Conversely, antagonistic activity was observed in two-thirds of the isolates when piperacillin/tazobactam was combined with minocycline. In addition, 40% of the isolates from patients treated with minocycline and trimethoprim-sulfamethoxazole combinations presented antagonistic activity, and 40% of the isolates from patients treated with rifampicin and piperacillin/tazobactam combinations presented antagonistic activity [50]. The clinical effectiveness of combination treatment for *Elizabethkingia* spp. infections has rarely been studied in the literature. Thus, this issue requires additional assessment by medical experts.

## 5. Limitations

The present meta-analysis has many limitations. Initially, randomized controlled trials were carried out by medical professionals, which is challenging because infections caused by *Elizabethkingia* spp. are rather uncommon. Twenty studies, all of which were retrospective in nature, were included in our analysis. This meta-analysis was limited by the substantial risk of bias present in all of the included trials. Second, this meta-analysis has the drawback that few cases of infections caused by *Elizabethkingia* spp. were included. Only six studies included more than 50 patients in total. Most studies were conducted in Eastern countries, while only four were conducted in Western countries. There were biases related to the small number of patients and their geographic location. Third, the absence of evidence-based information in the literature prevented us from investigating the mortality risk of comorbidities related to infections caused by *Elizabethkingia* spp. in great depth. Fourth, there is little data on antibiotic treatment in the literature, making it impossible to thoroughly evaluate the effects of antibiotic therapy, and the present meta-analysis cannot be used to draw conclusions about the risk of mortality in patients with infections caused by *Elizabethkingia* spp. who receive antibiotic treatment.

## 6. Conclusions

The present meta-analysis of 20 studies provides evidence that the risk factors for mortality in patients with infections caused by *Elizabethkingia* spp. are ICU admission, the need for mechanical ventilation, immunosuppressive or steroid therapy, pneumonia, comorbid liver disease (mainly liver cirrhosis), and the use of inappropriate antimicrobial therapy. The mortality rate was 30.1%. Patients with infections caused by *Elizabethkingia* spp. require prompt identification and effective antibiotic treatment since these spp. are typically resistant to multiple antibiotics and have variable susceptibility patterns.

## 7. Future Directions

Antimicrobial susceptibility testing would be a more reliable means of guiding treatment. The identification of the best antimicrobial drugs is needed to ensure optimal treatment recommendations for treating *Elizabethkingia*-related infections.

## Figures and Tables

**Table 1:** Characteristics of the 20 included studies.

Author/Year	Region	Study Design	No. of Total Patients	No. of Dead Patients
Figueroa CCE et al., 2017 [33]	USA	RET	11	2
Chang Y et al., 2021 [34]	China	RET	39	20
Choi MH et al., 2019 [19]	Korea	RET	30	10
Guerpillon B et al., 2022 [35]	France	RET	20	6
Hsu MS et al., 2011 [20]	Taiwan	RET	118	28
Hung PP et al., 2008 [22]	Taiwan	RET	30	10
Huang YC et al., 2018 [25]	Taiwan	RET	66	25
Joo HD et al., 2015 [36]	Korea	RET	30	9
Lau SK et al., 2016 [16]	Hong Kong	RET	17	3
Lin JN et al., 2018 [37]	Taiwan	RET	92	25
Lin YT et al., 2009 [24]	Taiwan	RET	32	13
Li Y et al., 2022 [38]	China	RET	24	10
Ma S et al., 2023 [21]	China	RET	92	34
Moore LS et al., 2016 [39]	UK	RET	30	5
Sarathi S et al., 2023 [14]	India	RET	80	21
Seong H et al., 2020 [40]	Korea	RET	210	53
Singh S et al., 2020 [41]	India	RET	18	3
Umair A et al., 2021 [42]	Pakistan	RET	10	5
Weaver KN et al., 2010 [43]	USA	RET	19	9
Yum JH et al., 2010 [44]	USA	RET	22	7

Footnotes: RET: retrospective study; USA: United States of America; UK: United Kingdom; No.: number.

**Table 2:** Risk bias of the included 20 studies.

Author/Year	Confounding	Selection	Interventions Classification	Interventions Deviations	Missing Data	Measurement of Outcomes	Selective Results
Figueroa CCE et al., 2017 [33]	Serious risk	Serious risk	Serious risk	Serious risk	Serious risk	Serious risk	Serious risk
Chang Y et al., 2021 [34]	Low risk	Moderate risk	Low risk	Low risk	Low risk	Moderate risk	Low risk
Choi MH et al., 2019 [19]	Moderate risk	Moderate risk	Low risk	Moderate risk	Low risk	Low risk	Low risk
Guerpillon B et al., 2022 [35]	Moderate	Moderate risk	Serious risk	Moderate risk	Moderate risk	Moderate risk	Moderate risk
Hsu MS et al., 2011 [20]	Low risk	Low risk	Low risk	Low risk	Low risk	Low risk	Low risk
Hung PP et al., 2008 [22]	Moderate risk	Moderate risk	Moderate risk	Moderate risk	Low risk	Low risk	Low risk
Huang YC et al., 2018 [25]	Low risk	Moderate risk	Moderate risk	Moderate risk	Moderate risk	Low risk	Low risk
Joo HD et al., 2015 [36]	Serious risk	Moderate risk	Moderate risk	Serious risk	Moderate risk	Moderate risk	Moderate risk
Lau SK et al., 2016 [16]	Serious risk	Serious risk	Serious risk	Moderate risk	Moderate risk	Moderate risk	Serious risk
Lin JN et al., 2018 [37]	Low risk	Low risk	Low risk	Low risk	Low risk	Low risk	Low risk
Lin YT et al., 2009 [24]	Low risk	Moderate risk	Low risk	Low risk	Low risk	Moderate risk	Low risk
Li Y et al., 2022 [38]	Moderate risk	Moderate risk	Moderate risk	Moderate risk	Moderate risk	Moderate risk	Serious risk
Ma S et al., 2023 [21]	Low risk	Low risk	Low risk	Low risk	Low risk	Low risk	Low risk
Moore LS et al., 2016 [39]	Serious risk	Serious risk	Moderate risk	Moderate risk	Moderate risk	Moderate risk	Serious risk
Sarathi S et al., 2023 [14]	Low risk	Low risk	Low risk	Moderate risk	Low risk	Low risk	Low risk
Seong H et al., 2020 [40]	Low risk	Low risk	Low risk	Low risk	Low risk	Low risk	Low risk
Singh S et al., 2020 [41]	Serious risk	Serious risk	Serious risk	Serious risk	Serious risk	Moderate risk	Serious risk
Umair A et al., 2021 [42]	Serious risk	Serious risk	Serious risk	Serious risk	Serious risk	Serious risk	Serious risk
Weaver KN et al., 2010 [43]	Serious risk	Serious risk	Serious risk	Serious risk	Serious risk	Serious risk	Serious risk
Yum JH et al., 2010 [44]	Serious risk	Moderate risk	Moderate risk	Serious risk	Serious risk	Serious risk	Serious risk

## Data Availability

The datasets generated during and/or analyzed during the current study are not publicly available but are available from the corresponding author upon reasonable request.

## Supplementary Materials

The following supporting information can be downloaded at: https://www.mdpi.com/article/10.3390/medicina60091529/s1, Figure S1: Mortality in patients between male and female; Figure S2: Mortality in patients between patients aged ≥65 years and those aged <65 years; Figure S3: Mortality in patients with and without meningitis; Figure S4: Mortality in patients with and without bacteraemia; Figure S5: Mortality in patients with and without cardiac disease; Figure S6: Mortality in patients with and without pulmonary disease; Figure S7: Mortality in patients with and without chronic kidney disease and ESRD; Figure S8: Mortality in patients with and without diabetes mellitus; Figure S9: Mortality in patients with and without cerebral vascular disease; Figure S10: Mortality in patients with and without malignant disease; Figure S11: Mortality in patients receiving piperacillin/tazobactam-based antibiotic therapy and not receive such therapy; Figure S12: Mortality in patients receiving fluoroquinolone-based antibiotic therapy and not receive such therapy; Figure S13: Mortality in patients receiving minocycline-based antibiotic therapy and not receive such therapy.
