# Supplementary material for: Mortality Risk and Antibiotic Therapy for Patients with Infections Caused by Elizabethkingia Species—A Meta-Analysis
Source: Medicina (Kaunas). 2024 Sep 19;60(9):1529. doi: 10.3390/medicina60091529 (PMC11433677; doi:10.3390/medicina60091529)

Figure S1. Mortality in patients between male and female [16,19–22,24,33–44].

Study or Subgroup	Male Events	Male Total	Female Events	Female Total	Weight	Risk Ratio M-H, Fixed, 95% CI
Chang Y, 2021	12	18	8	21	6.3%	1.75 [0.93, 3.30]
Choi MH, 2019	8	19	2	11	2.2%	2.32 [0.59, 9.02]
Figueroa CCE, 2017	1	5	1	6	0.8%	1.20 [0.10, 14.69]
Guerpillon B, 2022	2	12	4	8	4.1%	0.33 [0.08, 1.41]
Hsu MS, 2011	17	71	11	47	11.3%	1.02 [0.53, 1.99]
Hung PP, 2008	8	24	2	6	2.7%	1.00 [0.28, 3.54]
Joo HD, 2015	5	17	4	13	3.9%	0.96 [0.32, 2.87]
Lau SK, 2016	1	13	2	4	2.6%	0.15 [0.02, 1.29]
Lin JN, 2018	20	64	5	28	5.9%	1.75 [0.73, 4.19]
Lin YT, 2009	11	26	2	6	2.8%	1.27 [0.38, 4.29]
Li Y, 2022	3	11	7	13	5.5%	0.51 [0.17, 1.50]
Ma S. 2023	20	64	14	28	16.6%	0.63 [0.37, 1.05]
Moore LS, 2016	2	21	3	9	3.6%	0.29 [0.06, 1.43]
Seong H, 2020	31	129	22	81	23.0%	0.88 [0.55, 1.42]
Singh S, 2020	2	14	1	4	1.3%	0.57 [0.07, 4.80]
Umair A, 2021	2	5	3	5	2.6%	0.67 [0.18, 2.42]
Weaver KN, 2010	2	7	7	12	4.4%	0.49 [0.14, 1.74]
Yum JH, 2010	7	14	0	8	0.5%	9.00 [0.58, 139.50]
**Total (95% CI)**		**534**		**310**	**100.0%**	**0.95 [0.76, 1.17]**
Total events	154		98			

Heterogeneity: Chi² = 22.36, df = 17 (P = 0.17); I² = 24%
Test for overall effect: Z = 0.51 (P = 0.61)

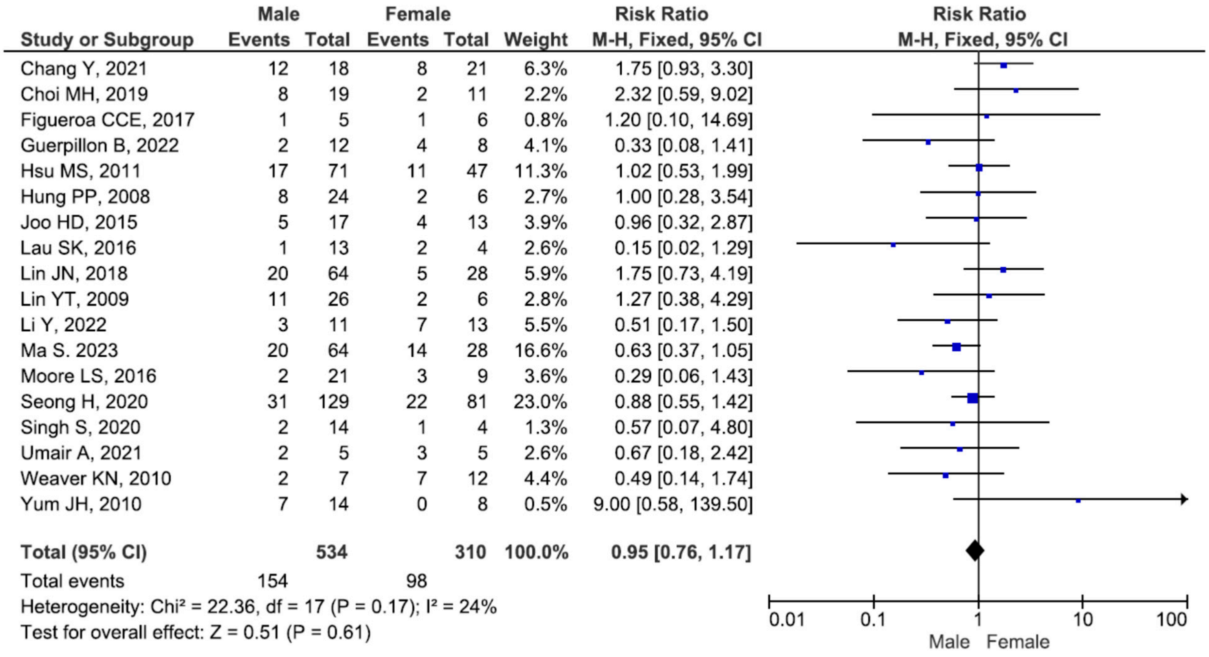

Figure S2. Mortality in patients between patients aged ≥65 years and those aged < 65 years [16,20,22,24,33,35,37,39,41–44].

Study or Subgroup	Age ≥65 years Events	Age ≥65 years Total	Control Events	Control Total	Weight	Risk Ratio M-H, Fixed, 95% CI
Figueroa CCE, 2017	1	7	1	4	2.6%	0.57 [0.05, 6.86]
Guerpillon B, 2022	6	19	0	1	1.9%	1.30 [0.11, 15.56]
Hsu MS, 2011	17	71	11	47	27.0%	1.02 [0.53, 1.99]
Hung PP, 2008	8	21	2	9	5.7%	1.71 [0.45, 6.54]
Lau SK, 2016	2	7	1	10	1.7%	2.86 [0.32, 25.72]
Lin JN, 2018	12	41	13	51	23.6%	1.15 [0.59, 2.24]
Lin YT, 2009	10	24	3	8	9.2%	1.11 [0.40, 3.06]
Moore LS, 2016	0	5	5	25	4.2%	0.39 [0.03, 6.20]
Singh S, 2020	1	8	2	10	3.6%	0.63 [0.07, 5.72]
Umair A, 2021	2	3	3	7	3.7%	1.56 [0.48, 5.02]
Weaver KN, 2010	4	11	5	8	11.8%	0.58 [0.23, 1.50]
Yum JH, 2010	3	7	4	15	5.2%	1.61 [0.48, 5.33]
**Total (95% CI)**		**224**		**195**	**100.0%**	**1.08 [0.78, 1.50]**
Total events	66		50			

Heterogeneity: Chi² = 4.72, df = 11 (P = 0.94); I² = 0%
Test for overall effect: Z = 0.47 (P = 0.64)

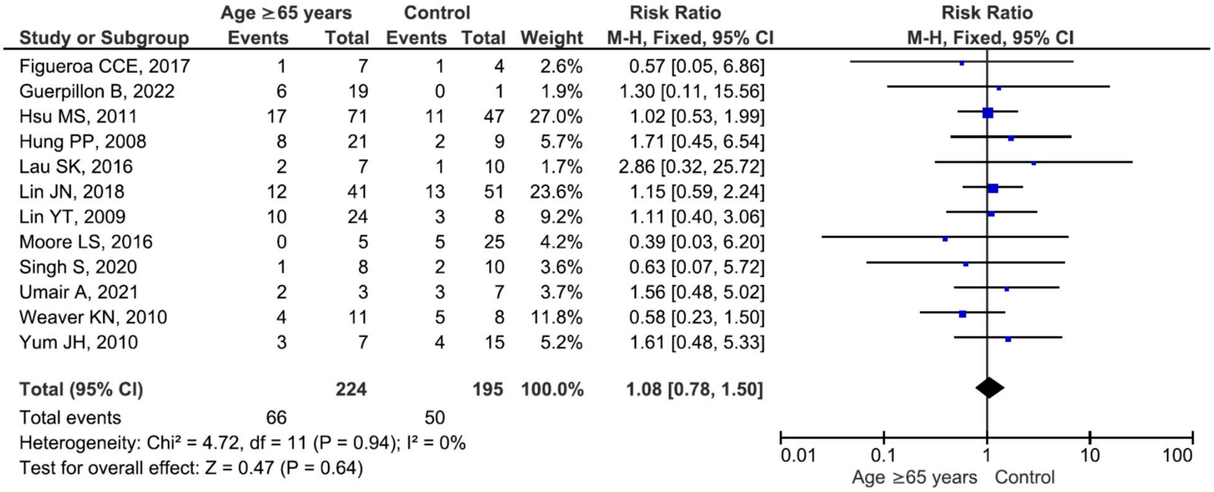

Figure S3. Mortality in patients with and without meningitis[22,24,34,41,42].

Study or Subgroup	Meningitis Events	Meningitis Total	Control Events	Control Total	Weight	Risk Ratio M-H, Fixed, 95% CI
Chang Y, 2021	1	3	19	36	35.6%	0.63 [0.12, 3.22]
Hung PP, 2008	0	1	10	29	16.0%	0.71 [0.06, 8.27]
Lin YT, 2009	1	1	12	31	17.9%	1.92 [0.77, 4.77]
Singh S, 2020	1	3	2	15	8.1%	2.50 [0.32, 19.53]
Umair A, 2021	0	1	5	9	22.3%	0.45 [0.04, 5.35]
**Total (95% CI)**		**9**		**120**	**100.0%**	**0.99 [0.46, 2.13]**
Total events	3		48			

Heterogeneity: Chi² = 3.57, df = 4 (P = 0.47); I² = 0%
Test for overall effect: Z = 0.03 (P = 0.98)

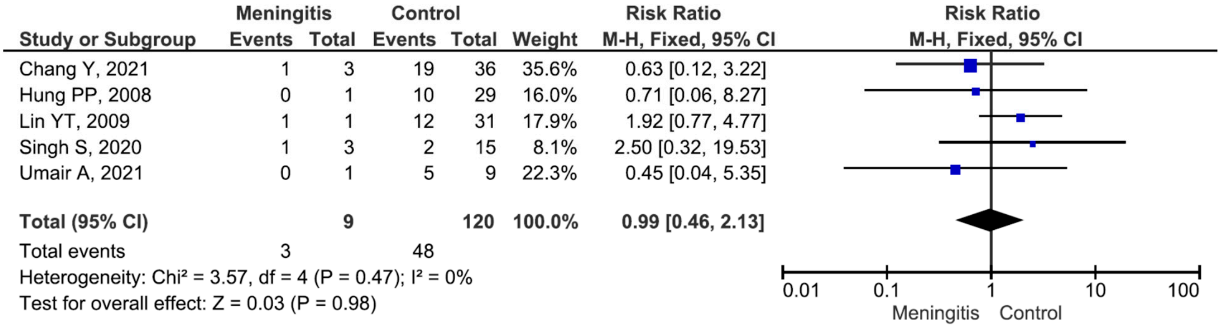

Figure S4. Mortality in patients with and without bacteraemia[16,20,22,24,33–35,39–44].

Study or Subgroup	Bacteremia Events	Bacteremia Total	Control Events	Control Total	Weight	Risk Ratio M-H, Fixed, 95% CI
Chang Y, 2021	3	6	17	33	10.9%	0.97 [0.41, 2.31]
Figueroa CCE, 2017	1	8	1	3	3.0%	0.38 [0.03, 4.27]
Guerpillon B, 2022	6	17	0	3	1.7%	2.89 [0.20, 41.49]
Hsu MS, 2011	20	91	8	27	25.8%	0.74 [0.37, 1.49]
Hung PP, 2008	7	17	3	13	7.1%	1.78 [0.57, 5.60]
Lau SK, 2016	2	12	1	5	3.0%	0.83 [0.10, 7.24]
Lin YT, 2009	10	22	3	10	8.6%	1.52 [0.53, 4.34]
Moore LS, 2016	1	1	4	29	1.2%	5.00 [1.55, 16.09]
Seong H, 2020	4	24	49	186	23.4%	0.63 [0.25, 1.60]
Singh S, 2020	2	9	1	9	2.1%	2.00 [0.22, 18.33]
Umair A, 2021	3	6	2	4	5.0%	1.00 [0.28, 3.54]
Weaver KN, 2010	2	3	7	16	4.6%	1.52 [0.58, 4.04]
Yum JH, 2010	1	3	6	19	3.4%	1.06 [0.19, 5.96]
**Total (95% CI)**		**219**		**357**	**100.0%**	**1.05 [0.75, 1.46]**
Total events	62		102			

Heterogeneity: Chi² = 12.47, df = 12 (P = 0.41); I² = 4%
Test for overall effect: Z = 0.27 (P = 0.79)

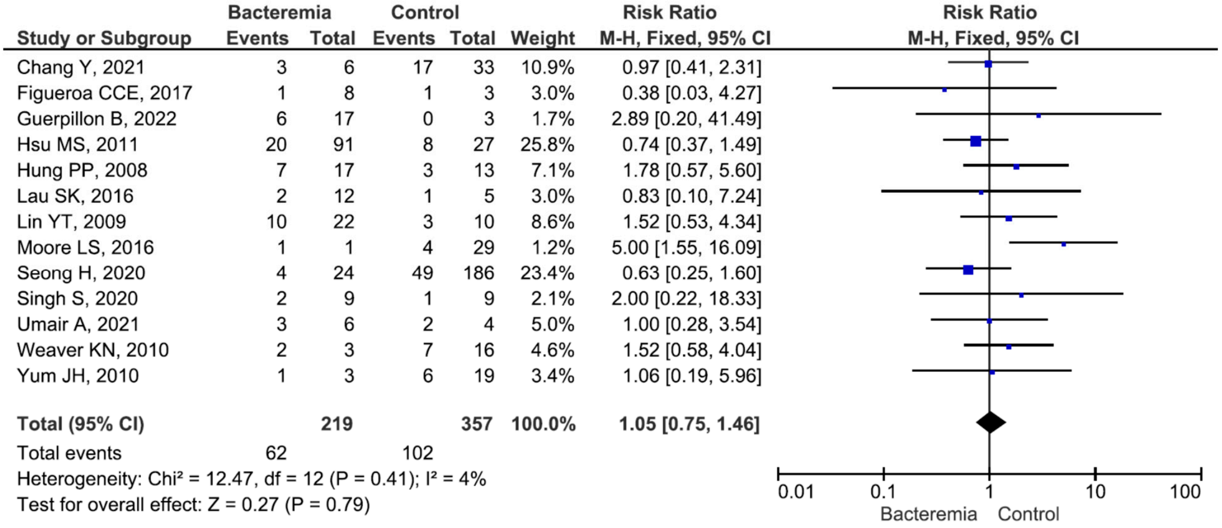

Figure S5. Mortality in patients with and without cardiac disease[14,16,20–22,40,42,44].

	Cardiac diseases		Control			Risk Ratio
**Study or Subgroup**	**Events**	**Total**	**Events**	**Total**	**Weight**	**M-H, Fixed, 95% CI**
Hsu MS, 2011	1	7	27	111	8.0%	0.59 [0.09, 3.71]
Hung PP, 2008	4	8	6	22	8.0%	1.83 [0.69, 4.85]
Lau SK, 2016	2	10	1	7	2.9%	1.40 [0.16, 12.60]
Ma S. 2023	4	7	30	85	11.4%	1.62 [0.80, 3.27]
Sarathi S. 2023	2	12	19	68	14.2%	0.60 [0.16, 2.24]
Seong H, 2020	16	55	37	155	48.4%	1.22 [0.74, 2.01]
Umair A, 2021	2	3	3	7	4.5%	1.56 [0.48, 5.02]
Yum JH, 2010	1	2	6	21	2.6%	1.75 [0.37, 8.18]
**Total (95% CI)**		**104**		**476**	**100.0%**	**1.21 [0.86, 1.70]**
Total events	32		129			

Heterogeneity: Chi² = 3.47, df = 7 (P = 0.84); I² = 0%
Test for overall effect: Z = 1.09 (P = 0.27)

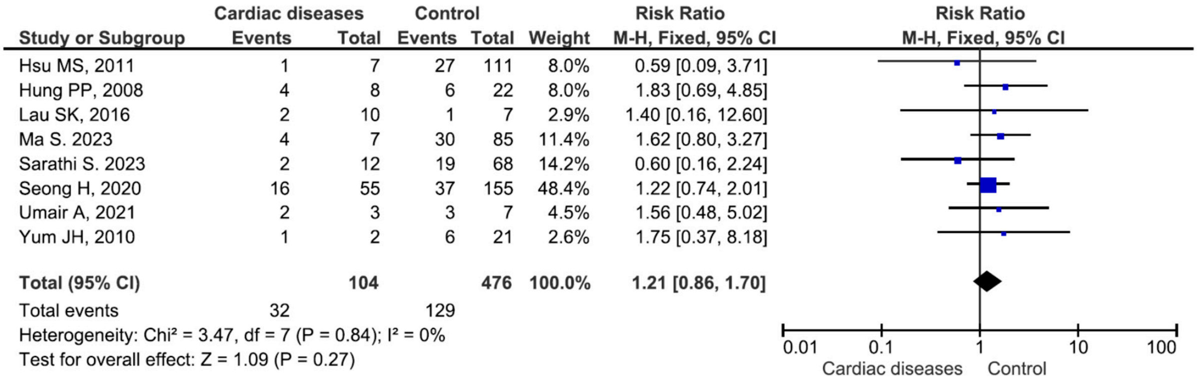

Figure S6. Mortality in patients with and without pulmonary disease[14,16,21,22,33,34,37,40,41].

	Pulmonary diseases		Control			Risk Ratio
**Study or Subgroup**	**Events**	**Total**	**Events**	**Total**	**Weight**	**M-H, Fixed, 95% CI**
Chang Y, 2021	19	30	1	9	3.8%	5.70 [0.88, 36.90]
Figueroa CCE, 2017	1	4	1	7	1.8%	1.75 [0.15, 21.00]
Hung PP, 2008	2	4	8	26	5.3%	1.63 [0.52, 5.07]
Lau SK, 2016	0	2	3	15	2.7%	0.76 [0.05, 11.27]
Lin JN, 2018	4	9	21	83	10.2%	1.76 [0.77, 3.98]
Ma S. 2023	1	9	33	83	16.1%	0.28 [0.04, 1.81]
Sarathi S. 2023	14	29	7	51	12.6%	3.52 [1.61, 7.71]
Seong H, 2020	9	43	44	167	44.8%	0.79 [0.42, 1.50]
Singh S, 2020	0	2	3	16	2.6%	0.81 [0.05, 12.01]
**Total (95% CI)**		**132**		**457**	**100.0%**	**1.40 [0.98, 2.01]**
Total events	50		121			

Heterogeneity: Chi² = 14.14, df = 8 (P = 0.08); I² = 43%
Test for overall effect: Z = 1.84 (P = 0.07)

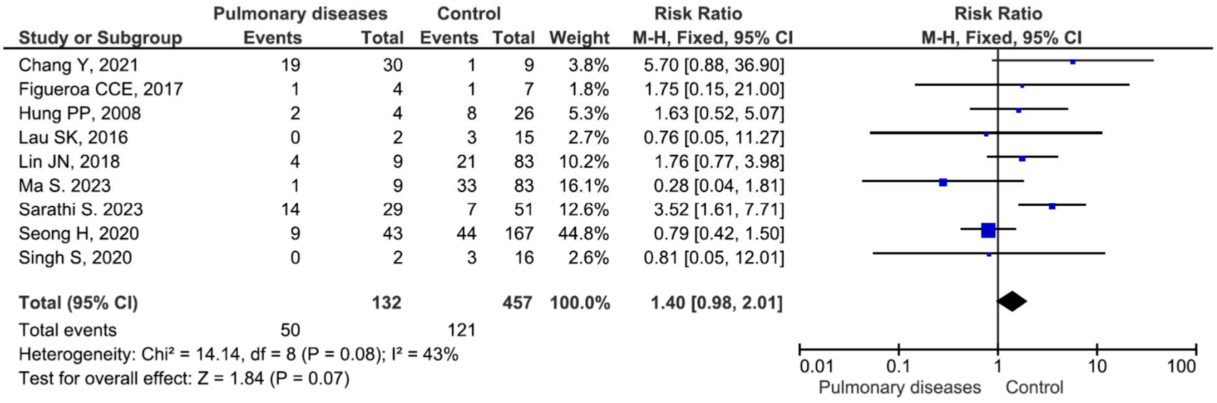

Figure S7. Mortality in patients with and without chronic kidney disease and ESRD [14,16,20,22,24,33,36–38,40–42].

Study or Subgroup	CKD and ESRD Events	CKD and ESRD Total	Control Events	Control Total	Weight	Risk Ratio M-H, Fixed, 95% CI
Figueroa CCE, 2017	1	6	1	5	2.2%	0.83 [0.07, 10.20]
Hsu MS, 2011	1	6	27	112	5.5%	0.69 [0.11, 4.26]
Hung PP, 2008	0	2	10	28	4.0%	0.46 [0.04, 6.05]
Joo HD, 2015	5	8	4	22	4.3%	3.44 [1.22, 9.69]
Lau SK, 2016	2	4	1	13	0.9%	6.50 [0.78, 54.30]
Lin JN, 2018	1	5	24	87	5.2%	0.72 [0.12, 4.32]
Lin YT, 2009	0	5	13	27	9.6%	0.17 [0.01, 2.52]
Li Y, 2022	3	8	7	16	9.4%	0.86 [0.30, 2.46]
Sarathi S. 2023	2	6	19	74	5.7%	1.30 [0.39, 4.29]
Seong H, 2020	18	69	35	141	46.2%	1.05 [0.64, 1.72]
Singh S, 2020	0	1	3	17	1.4%	1.29 [0.10, 16.93]
Umair A, 2021	0	2	5	8	5.5%	0.27 [0.02, 3.61]
**Total (95% CI)**		**122**		**550**	**100.0%**	**1.01 [0.72, 1.42]**
Total events	33		149			

Heterogeneity: Chi² = 11.96, df = 11 (P = 0.37); I² = 8%
Test for overall effect: Z = 0.07 (P = 0.95)

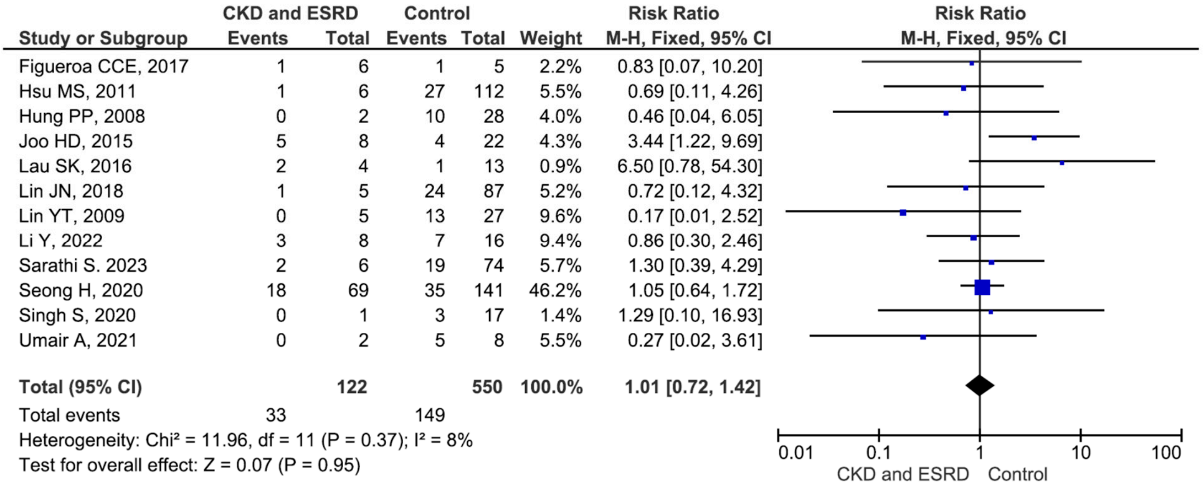

Figure S8. Mortality in patients with and without diabetes mellitus[14,16,20–22,33,37,38,40–42,44].

Study or Subgroup	Diabetes mellitus Events	Diabetes mellitus Total	Control Events	Control Total	Weight	Risk Ratio M-H, Fixed, 95% CI
Figueroa CCE, 2017	1	7	1	4	2.1%	0.57 [0.05, 6.86]
Hsu MS, 2011	6	30	22	88	18.6%	0.80 [0.36, 1.78]
Hung PP, 2008	4	9	6	21	6.0%	1.56 [0.57, 4.21]
Lau SK, 2016	0	4	3	13	3.1%	0.40 [0.02, 6.46]
Lin JN, 2018	8	24	17	68	14.8%	1.33 [0.66, 2.68]
Li Y, 2022	2	4	8	20	4.4%	1.25 [0.41, 3.82]
Ma S. 2023	3	7	31	85	7.9%	1.18 [0.48, 2.89]
Sarathi S. 2023	9	20	12	60	10.0%	2.25 [1.12, 4.53]
Seong H, 2020	9	41	44	169	28.6%	0.84 [0.45, 1.58]
Singh S, 2020	1	4	2	14	1.5%	1.75 [0.21, 14.69]
Umair A, 2021	5	6	0	4	1.0%	7.86 [0.55, 112.09]
Yum JH, 2010	0	1	7	21	2.1%	0.73 [0.06, 8.67]
**Total (95% CI)**		**157**		**567**	**100.0%**	**1.19 [0.90, 1.59]**
Total events	48		153			

Heterogeneity: Chi² = 8.78, df = 11 (P = 0.64); I² = 0%
Test for overall effect: Z = 1.22 (P = 0.22)

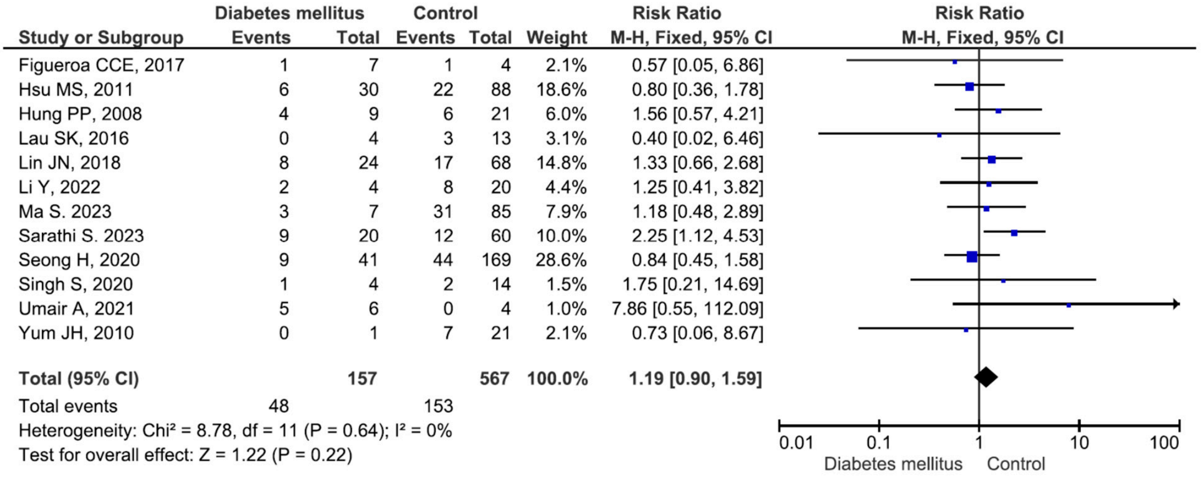

Figure S9. Mortality in patients with and without cerebral vascular disease[14,16,21,34,40,42].

Study or Subgroup	Cerebral vascular disease Events	Total	Control Events	Total	Weight	Risk Ratio M-H, Random, 95% CI
Chang Y, 2021	11	15	9	24	22.7%	1.96 [1.07, 3.56]
Lau SK, 2016	1	1	2	16	10.4%	5.10 [1.26, 20.61]
Ma S. 2023	17	32	17	60	24.4%	1.88 [1.12, 3.15]
Sarathi S. 2023	2	7	19	73	12.2%	1.10 [0.32, 3.77]
Seong H, 2020	8	47	45	163	21.2%	0.62 [0.31, 1.22]
Umair A, 2021	1	2	4	8	9.0%	1.00 [0.21, 4.71]
**Total (95% CI)**		**104**		**344**	**100.0%**	**1.47 [0.84, 2.56]**
Total events	40		96			

Heterogeneity: Tau² = 0.26; Chi² = 12.53, df = 5 (P = 0.03); I² = 60%
Test for overall effect: Z = 1.36 (P = 0.17)

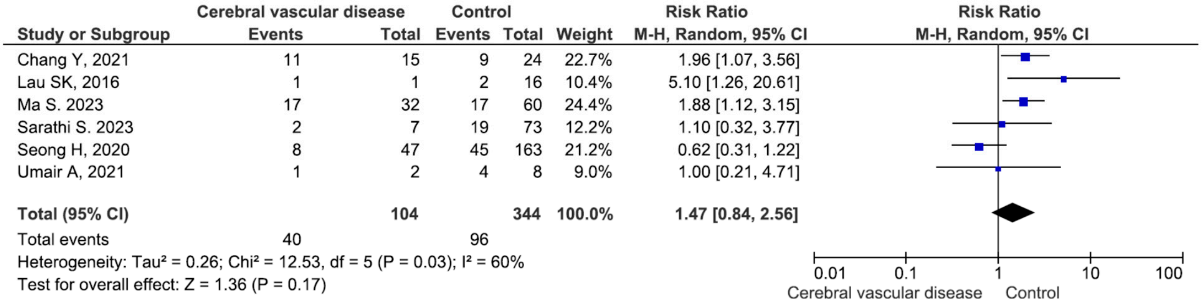

Figure S10. Mortality in patients with and without malignant disease[14,16,20–22,24,33,37,38,40–42,44].

Study or Subgroup	malignancies Events	Total	Control Events	Total	Weight	Risk Ratio M-H, Fixed, 95% CI
Figueroa CCE, 2017	0	5	2	6	2.7%	0.23 [0.01, 3.97]
Hsu MS, 2011	12	42	16	76	13.3%	1.36 [0.71, 2.59]
Hung PP, 2008	2	6	8	24	3.7%	1.00 [0.28, 3.54]
Lau SK, 2016	1	2	2	15	0.5%	3.75 [0.56, 24.91]
Lin JN, 2018	12	40	13	52	13.2%	1.20 [0.62, 2.34]
Lin YT, 2009	5	13	8	19	7.6%	0.91 [0.38, 2.17]
Li Y, 2022	4	6	6	18	3.5%	2.00 [0.84, 4.75]
Ma S. 2023	9	21	25	71	13.3%	1.22 [0.68, 2.19]
Sarathi S. 2023	0	6	21	74	4.3%	0.25 [0.02, 3.69]
Seong H, 2020	27	100	26	110	28.9%	1.14 [0.72, 1.82]
Singh S, 2020	0	7	3	11	3.3%	0.21 [0.01, 3.61]
Umair A, 2021	1	2	4	8	1.9%	1.00 [0.21, 4.71]
Yum JH, 2010	1	6	6	16	3.8%	0.44 [0.07, 2.96]
**Total (95% CI)**		**256**		**500**	**100.0%**	**1.09 [0.85, 1.39]**
Total events	74		140			

Heterogeneity: Chi² = 8.86, df = 12 (P = 0.72); I² = 0%
Test for overall effect: Z = 0.67 (P = 0.50)

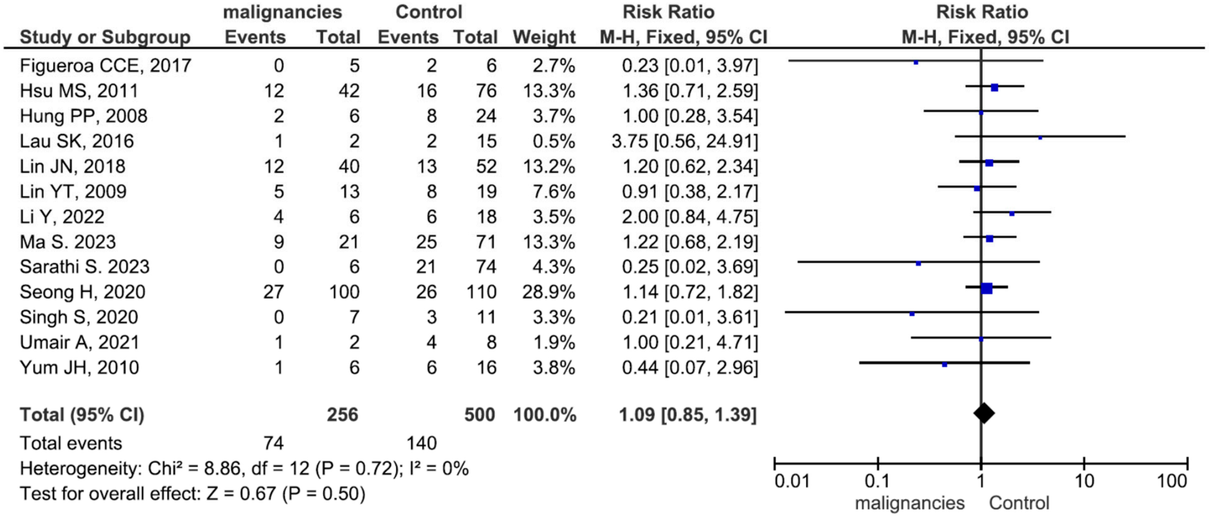

Figure S11. Mortality in patients receiving piperacillin/tazobactam-based antibiotic therapy and not receive such therapy[16,20,22,34,40].

Study or Subgroup	Anti-Pseudomonas penicillin-based therapy Events	Total	Control Events	Total	Weight	Risk Ratio M-H, Fixed, 95% CI
Chang Y, 2021	3	4	17	35	12.3%	1.54 [0.80, 2.99]
Hsu MS, 2011	3	10	25	108	15.0%	1.30 [0.47, 3.55]
Hung PP, 2008	0	4	10	26	11.6%	0.26 [0.02, 3.72]
Lau SK, 2016	0	2	3	15	3.9%	0.76 [0.05, 11.27]
Seong H, 2020	7	37	46	173	57.2%	0.71 [0.35, 1.45]
**Total (95% CI)**		**57**		**357**	**100.0%**	**0.85 [0.53, 1.37]**
Total events	13		101			

Heterogeneity: Chi² = 4.82, df = 4 (P = 0.31); I² = 17%
Test for overall effect: Z = 0.67 (P = 0.50)

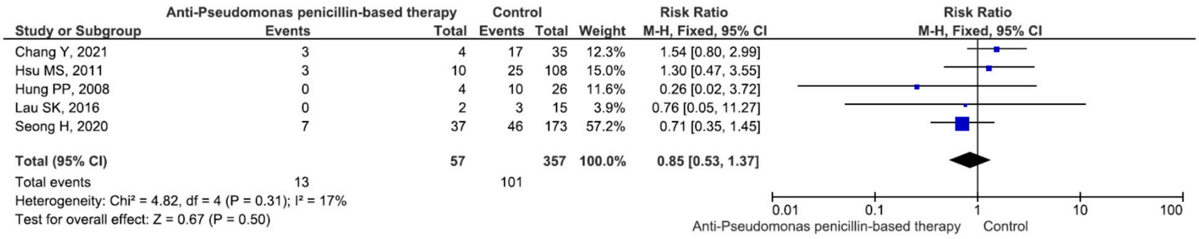

Figure S12. Mortality in patients receiving fluoroquinolone-based antibiotic therapy and not receive such therapy[16,19,20,22,25,34,40,42].

Study or Subgroup	Fluoroquinolone-based therapy Events	Total	Control Events	Total	Weight	Risk Ratio M-H, Fixed, 95% CI
Chang Y, 2021	3	7	17	32	9.1%	0.81 [0.32, 2.01]
Choi MH, 2019	5	9	5	21	4.5%	2.33 [0.89, 6.11]
Hsu MS, 2011	6	30	22	88	16.7%	0.80 [0.36, 1.78]
Huang YC, 2018	6	24	19	42	20.6%	0.55 [0.26, 1.19]
Hung PP, 2008	2	10	8	20	8.0%	0.50 [0.13, 1.93]
Lau SK, 2016	1	12	2	5	4.2%	0.21 [0.02, 1.81]
Seong H, 2020	18	69	35	141	34.3%	1.05 [0.64, 1.72]
Umair A, 2021	2	3	3	7	2.7%	1.56 [0.48, 5.02]
**Total (95% CI)**		**164**		**356**	**100.0%**	**0.88 [0.65, 1.18]**
Total events	43		111			

Heterogeneity: Chi² = 9.25, df = 7 (P = 0.24); I² = 24%
Test for overall effect: Z = 0.88 (P = 0.38)

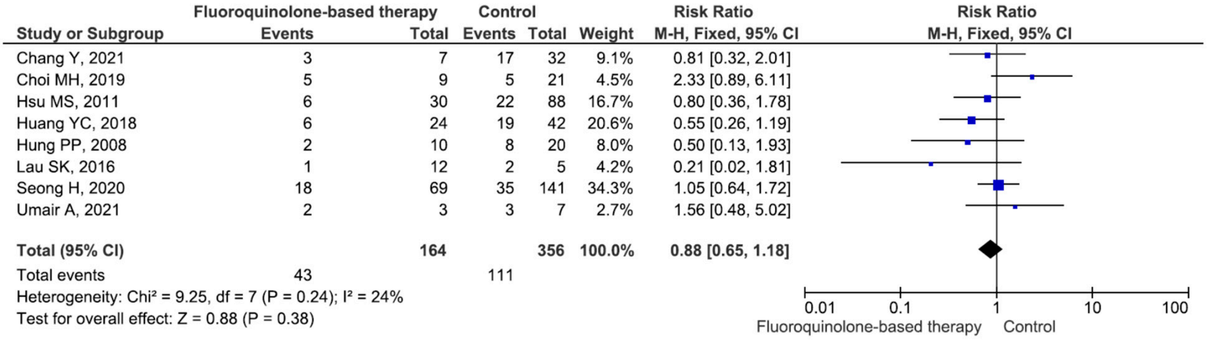

Figure S13. Mortality in patients receiving minocycline-based antibiotic therapy and not receive such therapy [34,42].

Study or Subgroup	Minocycline therapy Events	Total	Control Events	Total	Weight	Odds Ratio M-H, Fixed, 95% CI
Chang Y, 2021	3	4	17	35	59.2%	3.18 [0.30, 33.58]
Umair A, 2021	2	3	3	7	40.8%	2.67 [0.16, 45.14]
**Total (95% CI)**		**7**		**42**	**100.0%**	**2.97 [0.49, 18.13]**
Total events	5		20			

Heterogeneity: Chi² = 0.01, df = 1 (P = 0.93); I² = 0%
Test for overall effect: Z = 1.18 (P = 0.24)

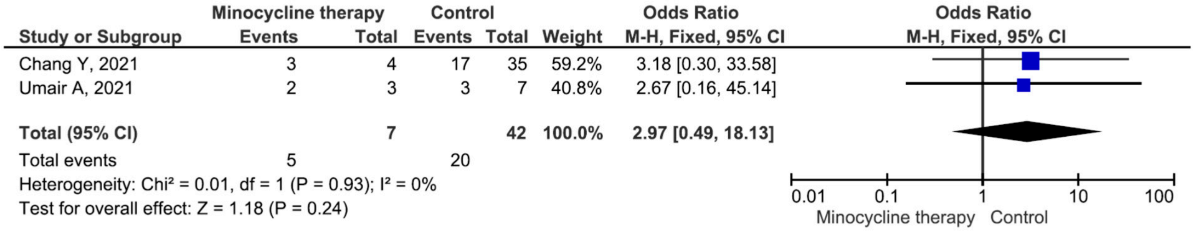

Supplement: Supplementary file 1 [file medicina-60-01529-s001.zip › medicina-3174191-supplementary.pdf]
